# Outcomes in provisional one-stent versus dedicated two-stent coronary bifurcation stenting techniques: a systematic review and meta-analysis

**DOI:** 10.1186/s43044-026-00757-0

**Published:** 2026-06-16

**Authors:** Harsh Agrawal, Pooja Dubey, Jay Tewari, Shubhechha Neupane, Vanshika Singh, Kendrick Shunk, Muditha Perera, Alok Kumar Dwivedi, Debabrata Mukherjee

**Affiliations:** 1 Washington Hospital Healthcare System, Fremont, USA; 2 Santa Clara University, Santa Clara, USA; 3 Department of Internal Medicine, Baptist Hospitals of Southeast Texas, Beaumont, USA; 4 King George’s Medical University, Lucknow, India; 5 San Francisco VA Medical Center, San Francisco, USA; 6 University of Missouri, Columbia, USA; 7 Texas Tech University Health Sciences Center El Paso, El Paso, USA

**Keywords:** Percutaneous coronary intervention, Bifurcation stenting, Complex PCI

## Abstract

**Background:**

The optimal long-term strategy for coronary bifurcation PCI remains debated. Earlier meta-analyses limited to long-term data suggested better outcomes with a provisional approach versus routine two-stent techniques.

**Methods:**

We conducted an updated, comprehensive meta-analysis of randomized controlled trials comparing provisional versus two-stent strategies. MEDLINE, Embase, and the Cochrane Library were searched through September 2025. Fifteen RCTs (n = 6978) met inclusion criteria. Using Stata 16.1, random-effects (DerSimonian–Laird) risk ratios (RRs) with 95% confidence intervals (CIs) were calculated for prespecified outcomes.

**Results:**

Relative risks (95% CIs) for provisional vs two-stent were: all-cause mortality 0.97 (0.72–1.30); cardiovascular mortality 0.98 (0.68–1.40); myocardial infarction 0.86 (0.62–1.19); target lesion revascularization 1.07 (0.80–1.44); stent thrombosis 1.36 (0.81–2.29); and MACE 1.27 (0.81–1.99). Across endpoints, pooled estimates did not show statistically significant differences between strategies.

**Conclusion:**

In this updated synthesis of randomized trials, the available evidence did not demonstrate clear superiority of either provisional or two-stent strategies for major clinical outcomes. Unlike prior long-term-only analyses, we did not observe higher mortality or myocardial infarction with two-stent approaches. Technique selection should remain individualized according to lesion anatomy, procedural complexity, and operator expertise. These findings should not be interpreted as proof of equivalence or non-inferiority.

*Trial registration* CRD420251167534.

**Supplementary Information:**

The online version contains supplementary material available at 10.1186/s43044-026-00757-0.

## Background

Clinically significant coronary bifurcation disease is not uncommon and presents unique challenges. Observational and trial data suggest that 15–20% of all lesions undergoing percutaneous coronary intervention (PCI) involve bifurcation coronary disease [1, 2] and are associated with poorer outcomes when compared to interventions on non-bifurcation lesions [3]. Early randomized controlled trials compared one-stent provisional and dedicated two-stent interventional techniques; initially presenting short-term data that favored provisional stenting or were equivocal [4–7]. Later trials were larger, examined different techniques such as double crush and T-and-protrusion, and included left main interventions [8–15]. Prior meta-analyses have compared one- and two-stent strategies; however, each included limited or sub-optimal data, such as observational data [16]. Two recent meta-analyses by Nairooz et al. and Ford et al. only used long-term data and found that provisional stenting was associated with lower all-cause mortality [17, 18]. Contemporary bifurcation PCI practice generally supports a provisional-first approach for most bifurcation lesions, while recognizing that selected complex bifurcations may require an upfront planned two-stent strategy. European Bifurcation Club consensus documents emphasize the importance of lesion anatomy, side-branch size and disease length, proximal optimization, final kissing balloon inflation when appropriate, and operator expertise in selecting and executing bifurcation PCI strategies. Therefore, pooled comparisons of provisional and two-stent approaches should be interpreted in the context of substantial clinical and procedural heterogeneity across trials. We aimed to perform an updated systematic review and meta-analysis of randomized controlled trials comparing provisional one-stent versus dedicated two-stent strategies for coronary bifurcation PCI.

## Methods

The study protocol was registered with PROSPERO under registration number CRD420251167534. This systematic review and meta-analysis was conducted in accordance with the Preferred Reporting Items for Systematic Reviews and Meta-Analyses (PRISMA) guidelines. MEDLINE, Embase, and the Cochrane Library were searched from inception through September 2025 for English-language, peer-reviewed randomized controlled trials comparing provisional one-stent and two-stent strategies for coronary bifurcation PCI. Search terms included combinations of “provisional,” “bifurcation,” “complex,” “one-stent,” “single-stent,” “two-stent,” “double-stent,” “DK crush,” “culotte,” “T-stenting,” “TAP,” “randomized,” and “randomized controlled trial.” Eligible studies were randomized controlled trials comparing a provisional one-stent strategy with an upfront or dedicated two-stent strategy in patients undergoing PCI for coronary bifurcation lesions. Trials comparing two different two-stent techniques without a provisional comparator, observational studies, conference abstracts without peer-reviewed full-text publication, and non-English articles were excluded.

All-cause mortality was the primary outcome of interest. Additional outcomes were major adverse cardiac events (MACE), myocardial infarction, target lesion revascularization, and stent thrombosis. Because only five studies directly reported all-cause mortality, we performed an exploratory mortality analysis in which cardiac death was used when all-cause mortality was unavailable. This approach was used only to maximize study-level information and should not be interpreted as a validated surrogate endpoint analysis, because cardiac mortality is not interchangeable with all-cause mortality and may underestimate non-cardiovascular deaths. Outcome definitions were accepted as reported by each individual trial. Definitions of myocardial infarction and MACE were not fully uniform across studies. MACE variably included combinations of death, cardiac death, myocardial infarction, target lesion revascularization, target vessel revascularization, stent thrombosis, or target lesion failure depending on the trial. Because individual patient-level data were unavailable, outcomes were pooled according to the definitions used in the original publications. This variability was considered an important source of clinical and methodological heterogeneity.

Two authors (HA and PD) independently extracted data on study population size, patient demographics, stenting strategies, and outcomes.

Relative risks (RR) with 95% confidence intervals were calculated using the Mantel–Haenszel method for each study outcome. Pooled effects with a 95% confidence interval for the outcomes were obtained using a random effects model, which used the DerSimonian and Laird method. In the case where a study contained a zero cell, 0.5 was added to derive finite variance estimators. A random effects model was used because the observed estimates of treatment effect can vary across studies due to real differences in treatment effect as well as differences in study populations. Higgins I^2^ statistic and the test of heterogeneity were used to assess heterogeneity between studies. An I^2^ > 50% was considered indicative of significant heterogeneity. Subgroup analyses were performed by follow-up duration, study size, and publication era when feasible. A formal technique-specific subgroup analysis by individual two-stent technique was considered but not performed because the number of trials per technique was small, several trials used mixed or protocol-dependent two-stent approaches, and procedural optimization practices differed substantially across studies. Therefore, pooled comparisons should not be interpreted as evidence that all two-stent techniques have equivalent efficacy or safety. Statistical significance level of ps was set at 0.05. Publication bias was assessed using funnel plots and Egger’s regression test when feasible. These assessments were interpreted cautiously because the number of studies was small for several endpoints, particularly MACE and directly reported all-cause mortality. Statistical analyses were conducted in Stata 16.1 (Stata Corp., College Station, TX, USA).

## Results

**Fig. 1:**
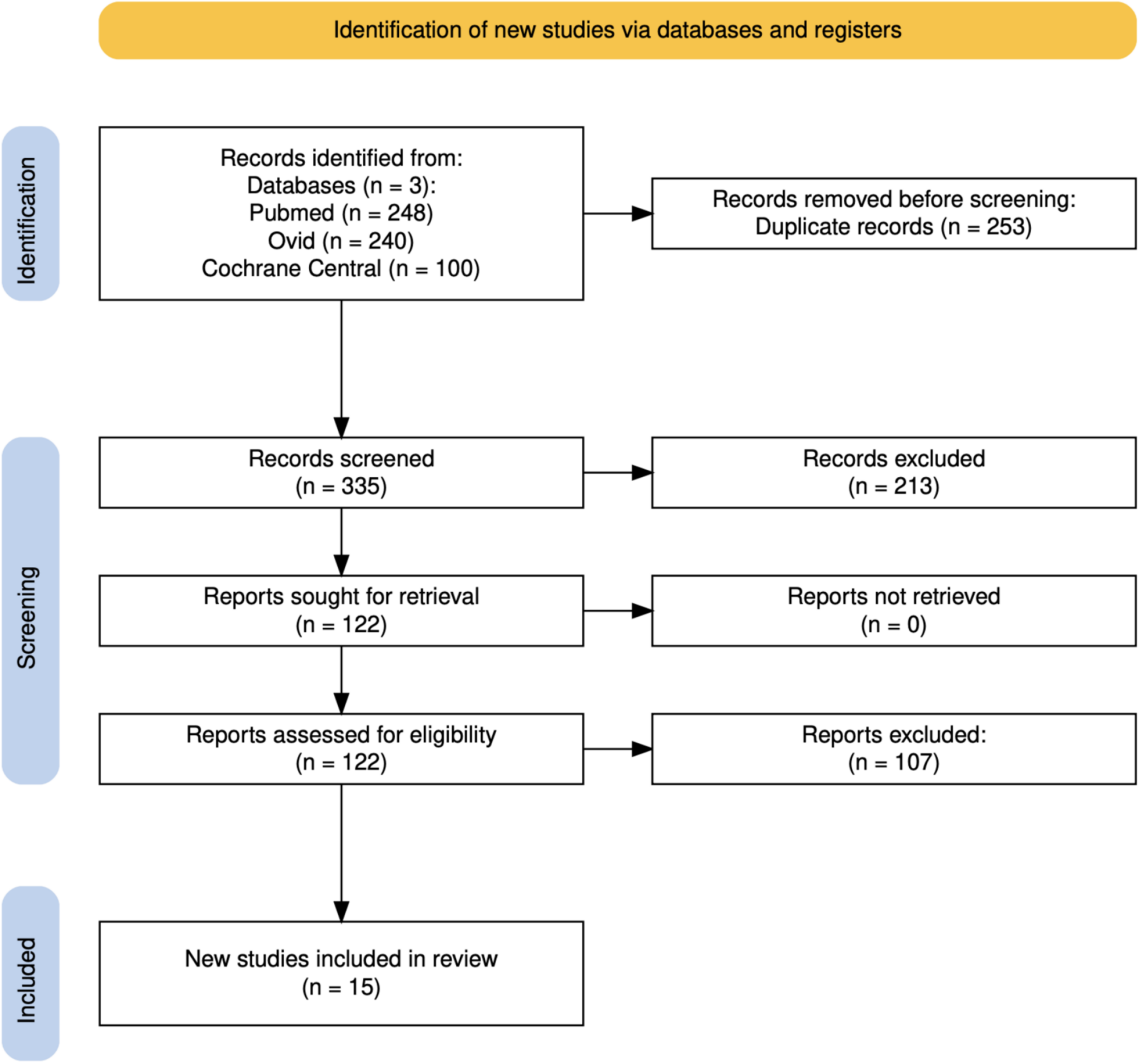
PRISMA study flow diagram

Fifteen randomized controlled trials met our inclusion criteria. The PRISMA study flow diagram has been shown in Fig. 1. A total of 6978 patients were enrolled in these studies: 4053 patients in the provisional strategy and 2925 patients in the two-stent strategy. The clinical follow-up time of these studies ranged from 6 to 60 months. Study and population characteristics are summarized in Table 1. Egger’s regression test did not show statistically significant evidence of small-study effects; however, this finding should be interpreted cautiously because the number of studies was limited for several endpoints.

**Table 1:** Study characteristics

Study	DKCRUSH-V	DKCRUSH-II	EBC two	Ferenc	BBS one	CACTUS	Colombo
Publication year	2017	2017	2016	2008	2009	2008	2003
Strategy	Provisional vs. DK crush	Provisional vs. DK crush	Provisional vs. culotte	Provisional vs. routine T-stenting	Simple vs. complex	Provisional vs. crush	Stent/PTCA vs. stent/stent
N	242|240	183|183	103|97	101|101	250|250	173|177	23|63
Follow up time (months)	12	60	12	12	9	6	6
Demographics							
Mean age (years) ± SD	64 ± 10|65 ± 9	64.7 ± 10|63.9 ± 11	62.9 ± 10.8|63.5 ± 12.1	66.7 ± 9.2|66.9 ± 10.5	64 ± 10|64 ± 11	67 ± 10|65 ± 10	62 ± 9|63 ± 10
Male (%)	78|83	79|79	85|78	79|78	77|77	76|80	91|76
Risk factors							
Diabetes mellitus (%)	25.6|28.8	23.1|19.6	25|31	25.7|18.8	13|11	22|23.7	26|21
Hyperlipidemia (%)	47.5|47.5	29.1|33.7	70|70		76|76	70.5|63.8	
Hypertension (%)	64.5|72.9	60.9|65.2	63|68	92.1|89.1	57|62	79.8|70.6	
Smoking (%)	32.2|34.2		56|50	9.9|13.9	17|17	16.8|20.3	
Renal dysfunction (%)	14.4|17.1	–	–	20.8|23.7	–	–	–
Medical history							
MI %	21.1|21.7	14.2|17.4	39|41	18.8|20.8	23|25	35.3|44.6	–
PCI %	17.8|13.8	20.9|21.2	40|41	44.6|51.5	17|16	26.6|31.1	–
CABG %	0.8|0.8	0.5|0	–	4|3		5.8|4.5	–
LVEF %	60 ± 9|59 ± 9	–	–	59 ± 12|61 ± 12	–	57|55	–
Clinical presentation							
Silent ischemia (%)	4.1|2.9	3.8|1.6	–	–	–	13.3|17.5	–
Stable angina (%)	10.4|14.2	11|15.3	69|68	–		36.4|31.1	–
Unstable angina (%)	74.4|70	68.7|66.8	–	–	11|13	47.4|44	17|17
Procedural characteristics							
Transradial access (%)	74.8|77.9	–	63|57	–	34|29	–	–
GP IIb/IIIa (%)	19|21.7	–	4|7	–	28|44	17.3|22.6	36.4|42.9
Kissing balloon inflation performed (%)	78.9|99.6	–	94|96	100|100	29|76	90.2|92.1	81.8|90.5
Stent diameter mm ± SD	3.29 ± 0.38|3.32 ± 0.37	2.79 ± 0.48|2.87 ± 0.49	3.06 ± 0.32|3.03 ± 0.33	–	3|3.2	–	–
LM segment length mm ± SD	28.8 ± 10.4|27.9 ± 9.9	28.8 ± 13.5|28.6 ± 12.4	23.4 ± 4.8|22.9 ± 5.1	21.7 ± 7.5|20.9 ± 8.2	–	–	–
Side Branch diameter mm ± SD	2.97 ± 0.38|2.92 ± 0.35	–	2.61 ± 0.29|2.72 ± 0.25	–	–	–	–
Side branch/second-vessel length mm ± SD	21.25 ± 7.44|21 ± 7.32	–	19.9 ± 6.8|20.7 ± 5.5	10.4 ± 4.1|9.9 ± 4.2	–	–	–
Number of vessels affected (%)							
1	–	34.6|30.4	76|65	34.7|25.8	69|73	–	–
2	–	–	17|29	27.7|26.7	25|22	–	–
3	–	–	6|5	37.6|47.5	6|5	–	–
Location of bifurcation (%)	–	–	–	–	–	–	–
LAD/D1	59.9|58.8	58.8|60.9	78|77	75.2|73.3	81|84	–	–
LCx/OM	48.8|50.4	16.5|12.5	15|19	15.8|20.8	14|11	–	–
RCA/PDA/PLV	64.5|62.5	8.8|9.2	6|4	8.9|5.9	4|5	–	–
Medina classification (%)	–	–	–	–	–	–	–
1,1,1	78.5|85	78.7|84.2	81|68	35.6|30.7	60|60	–	–
1,1,0	–	–	–	11.9|9.9	10|8	–	–
1,0,1	–	–	6|7	7.9|5.9	8|10	–	–
1,0,0	–	–	–	3|2	4|5	–	–
0,1,1	21.5|15	21.3|15.8	12|24	24.8|31.7	13|14	–	–

Study	Cross	Perfect	Nordic	Pan	Smart strategy	Definition II	EBC main	Crossover x SB opening
Publication year	2015	2015	2013	2004	2016	2020	2021	2021
Strategy	Control vs. experimental	Control vs. experimental	Main vessel vs. Main vessel + side branch	Simple vs. complex	Conservative vs. aggressive	Provisional vs. two stent	Provisional vs. dual stent	Simple crossover vs. SB opening
N	155|151	206|213	202|202	47|44	128|130	325|328	230|237	1685|509
Follow up time (months)	8	8	60	6	36	12	12	60
Demographics								
Mean age (years) ± SD	61 ± 7.9|61 ± 9.2	61.1 ± 8.8|60.9 ± 8.9	63 ± 10|63 ± 10	61 ± 10|58 ± 11	61.8 ± 10.1|61.5 ± 10.2	64 ± 10|63 ± 11	70.8 ± 10.1|71.4 ± 9.8	63.4 ± 11.1|63.7 ± 10.6
Male (%)	67|71	75|75	76|78	72|86		77|78	79|74	76|79
Risk factors								
Diabetes mellitus (%)	29|30.5	29.1|25.8	13|12	42|39	28.9|25.4	116 (35.7)|112 (34.1)	66 (29%)|62 (27%)	562 (33.4)|167 (32.8)
Hyperlipidemia (%)	49.7|47	57.3|62	78|72	53|41	12.5|13.1	223 (68.6)|227 (69.2)	158 (70%)|166 (72%)	628 (37.3)|207 (40.7)
Hypertension (%)	58.7|55.6	55.3|55.4	54|58	59|57	54.7|57.7	230 (70.1)|215 (66.2)	180 (79%)|190 (82%)	961 (57.0)|289 (56.8)
Smoking (%)	25.2|33.1	32.5|25.4		38|52	25.8|17.7	98 (30.2)|93 (28.4)	36 (16%)|30 (13%)	
Renal dysfunction (%)	0|2.6	0.5|0.5	–	–	1.6|3.1	48 (14.8)|59 (18)	12 (5%)|9 (4%)	527 (31.3)|152 (29.9)
Medical history								
MI %	–	–	–	–	7|7.7	12.9|11.9	26|28	
PCI %	–		25|25	–	10.9|6.9	16.6|19.8	41|43	11.1|13.8
CABG %	–		4|3	–	0|0.8	0.6|0		
LVEF %	62.2 ± 5.7|60.9 ± 7.0	59.5 ± 7.2|60.4 ± 6.8	–	60 ± 11|55 ± 11	60.5 ± 7.3|59.3 ± 10.7	60 ± 10|59 ± 10		58.43 ± 10.17|59.41 ± 8.77
Clinical presentation								
Silent ischemia (%)	–	–	2|1	–	10.2|5.4	5.2|5.2	10.9|13.5	
Stable angina (%)	54.2|49	62|61.3	66|65	–	62.5|63.1	21.8|24.1	13.5|8	37.7|42.0
Unstable angina (%)	40|43.7	31.7|34.9	32|34	–	20.3|23.8	50.5|48.8	3.5|3.0	62.3|58.0
Procedural characteristics								
Transradial access (%)	35.5|37.1	12.1|11.7	–	–	–	80.6|78.7	71|70	
GP IIb/IIIa (%)	–	–	51|51	–	–	14.8|18.3	5|4	
Kissing balloon inflation performed (%)	–	–	32|74	60|77	–		89|6	0|76.8
Stent diameter mm ± SD	3.3 ± 0.3|3.5 ± 2.2	3.3 ± 0.3|3.3 ± 0.3		2.9 ± 0.3|2.9 ± 0.3	3.3 ± 0.4|3.3 ± 0.4	3.02 ± 0.32|3.05 ± 0.32	3.8 ± 0.5|3.6 ± 0.6	3.34 ± 0.62|3.34 ± 0.62
LM segment length mm ± SD	33.0 ± 14.8|33.2 ± 13.1	36.9 ± 15.3|37.3 ± 14.7	23 ± 10|23 ± 10	25 ± 12|26 ± 9	24.9 ± 5.6|25.1 ± 5.3		22.1 ± 7|22.1 ± 7	
Side Branch diameter mm ± SD	2.8|2.6 ± 0.1	2.7 ± 0.2|2.7 ± 0.2	–	–	2.8 ± 0.2|2.9 ± 0.4	2.76 ± 0.38|2.76 ± 0.38	3.5 ± 0.6|3.5 ± 0.6	
Side branch/second-vessel length mm ± SD	30|24.7 ± 2.9	21.5 ± 6.9|21.4 ± 6.7	–	–	18.4 ± 7.8|17.7 ± 5.6		17.6 ± 6.9|19.3 ± 6.7	
Number of vessels affected (%)								
1	70.3|73.5	70.4|74.6	–	–	–			
2	27.7|23.2	25.2|21.6	–	–	–			
3	1.9|3.3	4.4|3.8	–	–	44.5|43.8			
Location of bifurcation (%)	–	–	–	–	–			
LAD/D1	88.4|90.7	92.2|93.9	0.7|0.7	77|80	7|3.1	60.6|62.5	77|77	47.2|47.3
LCx/OM	7.7|7.3	7.3|4.7	0.2|0.2	17|13	–	7.7|5.2	23|23	16.4|9.8
RCA/PDA/PLV	3.9|2	0.5|1.4	0.1|0.1	6|7	–	2.8|3.7		7.4|4.9
Medina classification (%)	–	–	–	–	–			
1,1,1	15.7|18.9	62.4|65.9	–	–	6.3|3.1	82.5|86.3	90|89	23.1|42.6
1,1,0	48.4|35.1	10.9|2.4	–	–	1.6|1.5			20.2|12.8
1,0,1	2.6|5.4	8.9|8.7	–	–	4.7|6.9			5.8|7.5
1,0,0	9.8|12.2	2|1	–	–	20.3|14.6			15.6|5.1
0,1,1	2.6|4.1	12.4|18.8	–	–	0.8|0.8	14.5|12.5	10|11	6.7|8.3

^*^Values reported as provisional | two stent; SD, standard deviation; Kissing balloon inflation timing varied across trials and may refer to first-stent, final, or post-second-stent kissing balloon inflation depending on the original study report

Length variables were reported inconsistently across trials and may refer to lesion length or stent length depending on the original study.

**Fig. 2:**
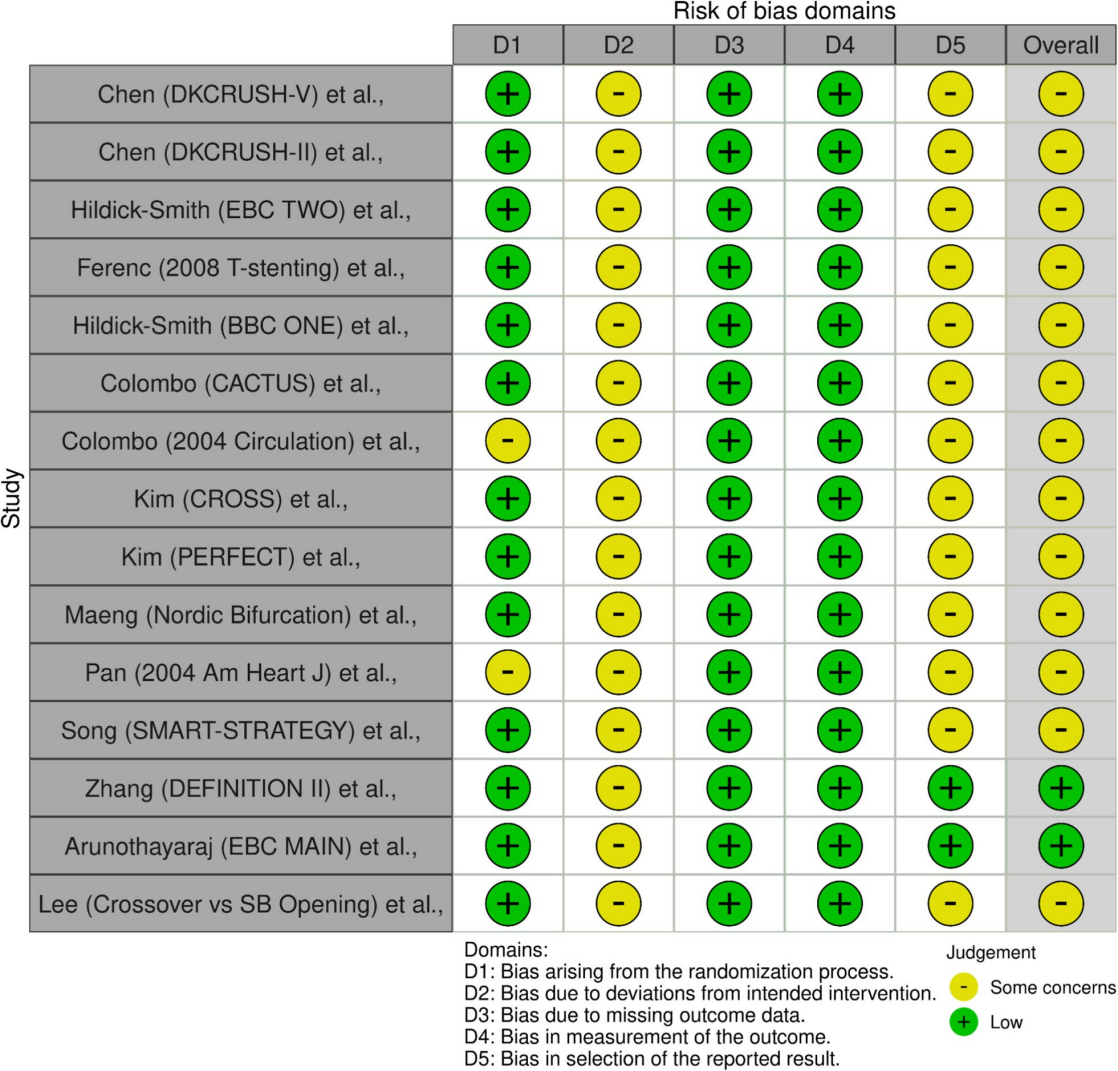
Risk of bias traffic light plot

**Fig. 3:**
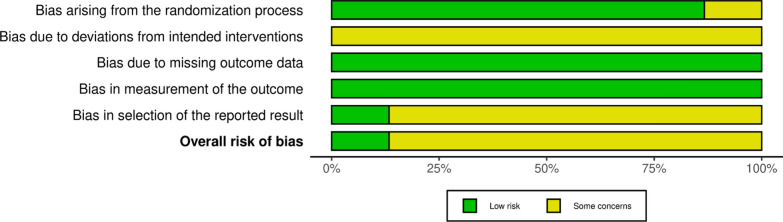
Risk of bias summary plot

The risk of bias assessment was done using the Revised Risk of Bias Tool for Randomized Trials (RoB2). The ROB traffic light plot (Fig. 2) and summary plot (Fig. 3) were generated using the Robvis tool.

### All-cause mortality

**Fig. 4:**
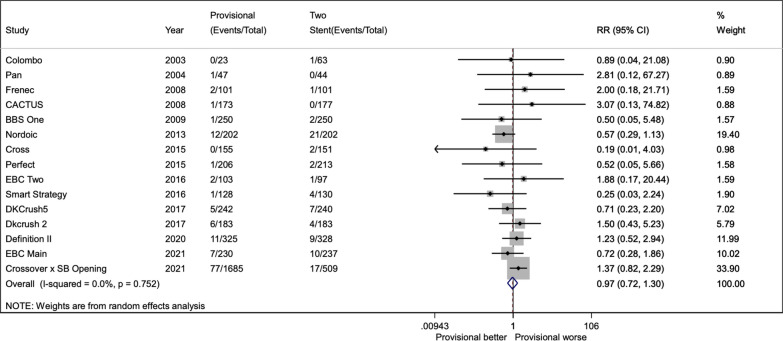
Forest plot for the estimated risks for all-cause mortality

Figure 4 shows the estimated risks for each study for all-cause mortality.

#### Primary analyses

Only five studies directly reported all-cause mortality. For the exploratory mortality endpoint, in which cardiac death was used when all-cause mortality was unavailable, pooled estimates did not show a statistically significant difference between provisional and two-stent strategies (RR, 0.97; 95% CI 0.72–1.30; p = 0.82; I^2^ = 0.0%). Pooled estimates also did not show statistically significant differences for cardiac death (RR, 0.98; 95% CI 0.68–1.40; p = 0.89), myocardial infarction (RR, 0.86; 95% CI 0.62–1.19; p = 0.36), target lesion revascularization (RR, 1.07; 95% CI 0.80–1.44; p = 0.64), stent thrombosis (RR, 1.36; 95% CI 0.81–2.29; p = 0.25), or MACE (RR, 1.27; 95% CI 0.81–1.99; p = 0.29). These non-significant findings should not be interpreted as evidence of equivalence or non-inferiority.

Heterogeneity varied by outcome. No meaningful heterogeneity was observed for the exploratory mortality endpoint, cardiac death, or stent thrombosis. In contrast, heterogeneity was moderate to substantial for myocardial infarction (I^2^ = 49.3%), target lesion revascularization (I^2^ = 44.0%), and MACE (I^2^ = 71.4%). This likely reflects differences across trials in lesion complexity, left main involvement, bifurcation anatomy, side-branch size and disease length, stenting technique, generation of drug-eluting stents, follow-up duration, and use of procedural optimization techniques.

Subgroup analyses: Subgroup analyses by follow-up duration and study size are summarized in Table 2. These analyses did not show statistically significant differences between provisional and two-stent strategies across the evaluated outcomes. Because stent platforms and bifurcation PCI techniques evolved over time, we also explored publication era as a pragmatic proxy for procedural and technological evolution. Studies published before 2015 showed a lower risk of myocardial infarction with provisional stenting compared with two-stent strategies (RR, 0.57; 95% CI 0.38–0.84; p = 0.004; I^2^ = 2.2%), whereas later studies did not show a statistically significant difference. This suggests that older trials and earlier procedural techniques may have contributed to heterogeneity in myocardial infarction outcomes. Sensitivity analyses also suggested that individual studies contributed to heterogeneity in MACE estimates; therefore, these findings should be interpreted cautiously.

**Table 2:** Subgroup analyses for the exploratory mortality endpoint and secondary outcomes

Characteristic	# of RCTs	RR (95% CI)					
		All-cause mortality	Cardiac deaths	MI	TLR	ST	MACE
*Follow-up duration (months)*							
Less than or equal to 12	4	0.926	0.985	0.939	1.141	1.361	1.168
		(0.572, 1.498)	(0.582, 1.667)	(0.631, 1.396)	(0.743, 1.751)	(0.724, 2.560)	(0.641, 2.129)
Greater than 12	11	0.911	0.967	0.609	0.997	1.359	1.517
		(0.479, 1.731)	(0.593, 1.577)	(0.354, 1.049)	(0.638, 1.559)	(0.546, 3.386)	(0.995, 2.313)
*Study size*							
Less than or equal to 300	5	1.047	0.903	0.622	0.866	0.682	1.172
		(0.333, 3.293)	(0.264, 3.086)	(0.311, 1.244)	(0.535, 1.400)	(0.188, 2.479)	(0.481, 2.856)
Greater than 300	10	0.96	0.982	0.912	1.122	1.555	1.247
		(0.703, 1.310)	(0.675, 1.428)	(0.623, 1.333)	(0.781, 1.611)	(0.881, 2.742)	(0.857, 1.816)
Overall	15	0.966	0.975	0.859	1.073	1.36	1.277
		(0.715, 1.304)	(0.682, 1.395)	(0.618, 1.193)	(0.798, 1.443)	(0.809, 2.287)	(0.829, 1.966)

RR, relative risk; CI, confidence interval; MI, myocardial infarction; TLR, target lesion revascularization; ST, stent thrombosis; MACE, major adverse cardiac event

### Cardiac deaths

**Fig. 5:**
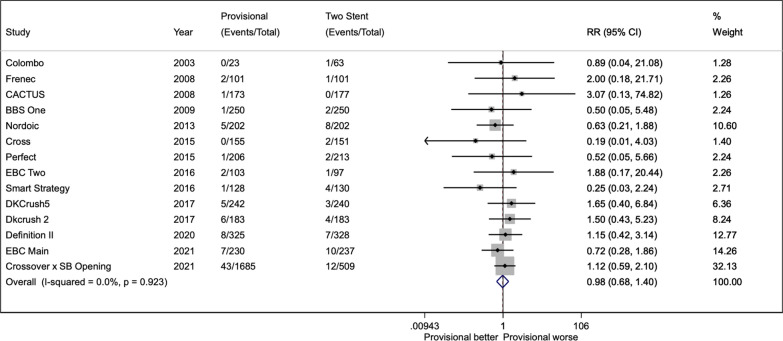
Forest plot for the estimated risks for cardiac death

Figure 5 shows the estimated risks for cardiac deaths. Data on cardiac deaths were available from fourteen studies. There was no significant difference in risk for cardiac deaths among the two treatment groups (pooled RR, 0.98, 95% CI 0.68–1.40, p = 0.891). I^2^ was 0.0% and the corresponding p for the test of heterogeneity was 0.923, providing no significant evidence of heterogeneity among the studies.

### Myocardial infarction

**Fig. 6:**
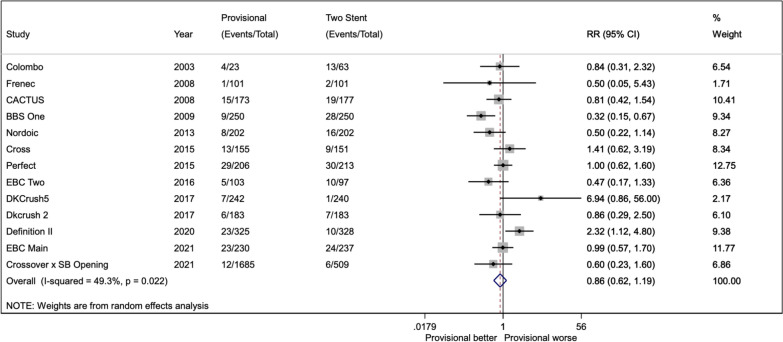
Forest plot for the estimated risks for myocardial infarction

Figure 6 shows the estimated risks for myocardial infarction. Thirteen studies reported data on myocardial infarction. The pooled relative risk for MI was 0.86 (95% CI 0.62–1.19) with a p = 0.363, indicating that there is no significant difference in MI between the two strategies. I^2^ was 49.3% with a p = 0.022 for the test of heterogeneity, providing significant evidence of heterogeneity among the studies.

### Target lesion revascularization

**Fig. 7:**
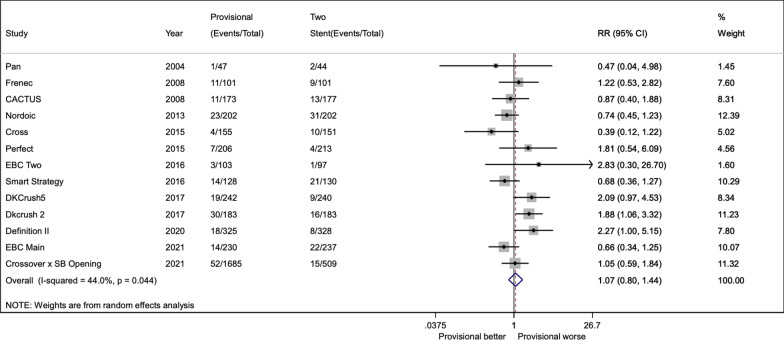
Forest plot for the estimated risks for target lesion revascularization

Figure 7 shows the estimated risks for target lesion revascularization. Data on TLR were available from thirteen studies. There was no significant difference in risk for TLR among the two treatment groups (pooled RR, 1.07, 95% CI 0.80–1.44, p = 0.642). The p for the test of heterogeneity was 0.044, and the corresponding I^2^ was 44.0% providing borderline evidence of heterogeneity among the studies.

### Stent thrombosis

**Fig. 8:**
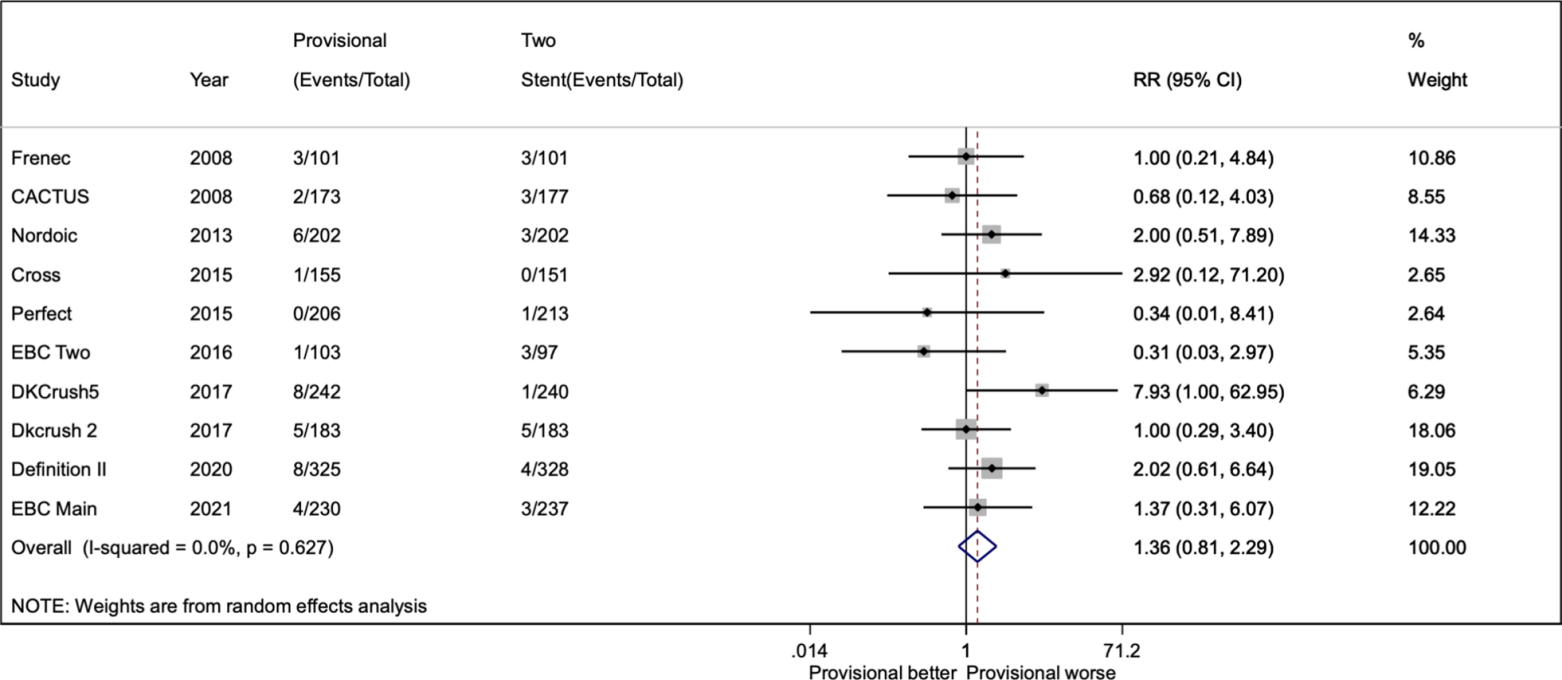
Forest plot for the estimated risks for stent thrombosis

Figure 8 shows the estimated risks for stent thrombosis. Ten studies out of the fifteen trials reported ST data. The pooled relative risk for ST was 1.36 (95% CI 0.81–2.29) with a p = 0.246. This indicates that there is no statistically significant difference between the provisional and the two-stent techniques for ST in each of the studies. There was no significant evidence of heterogeneity among the studies (I^2^ = 0.0%, p = 0.627).

### Major adverse cardiac events

**Fig. 9:**
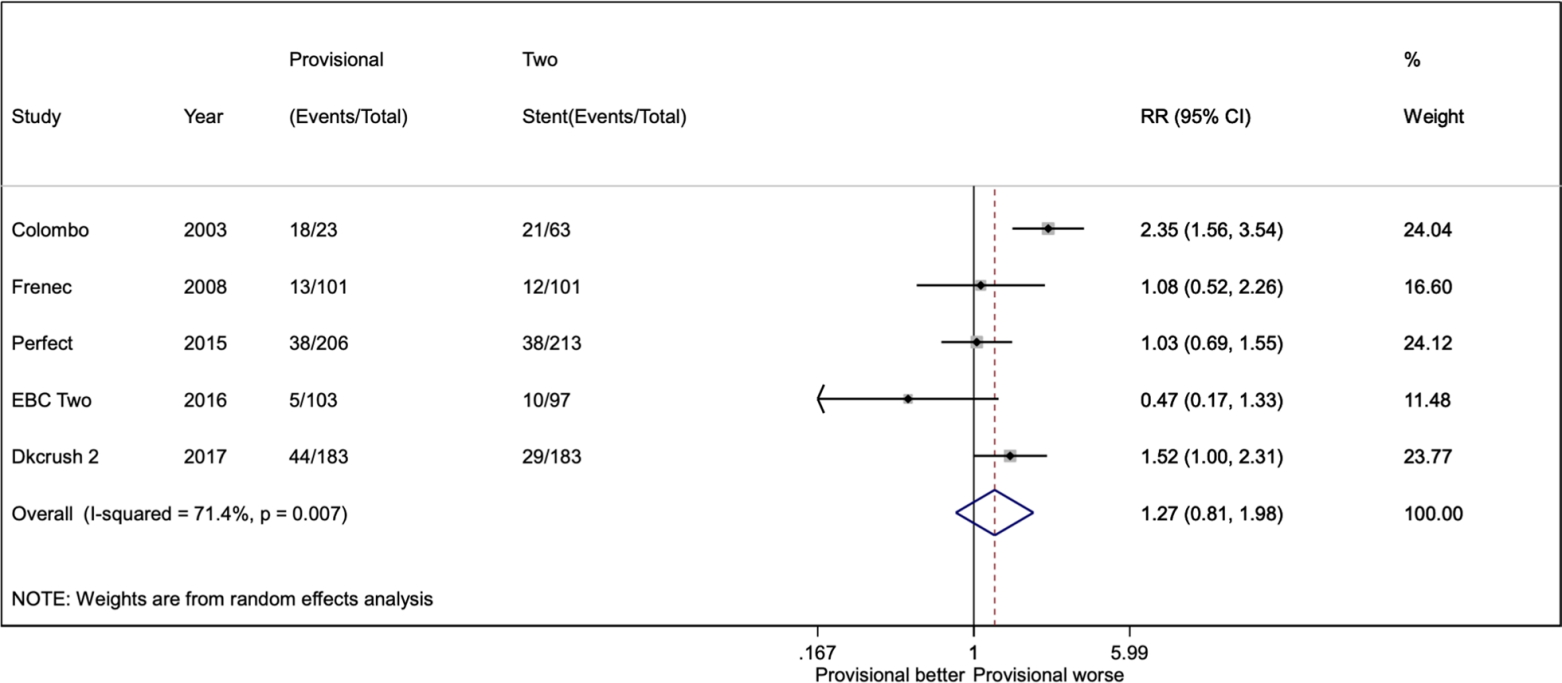
Forest plot for the estimated risks for major adverse cardiac events

Figure 9 shows the estimated risks for major adverse cardiac events. Data from only five studies were available on MACE. The pooled relative risk for MACE was 1.27 (95% CI 0.81 to 1.99) with a p = 0.294, indicating that there is no significant difference in MACE between the two strategies. Significant evidence of heterogeneity among the studies regarding MACE was observed (I^2^ = 71.4%, p = 0.007).

**Fig. 10:**
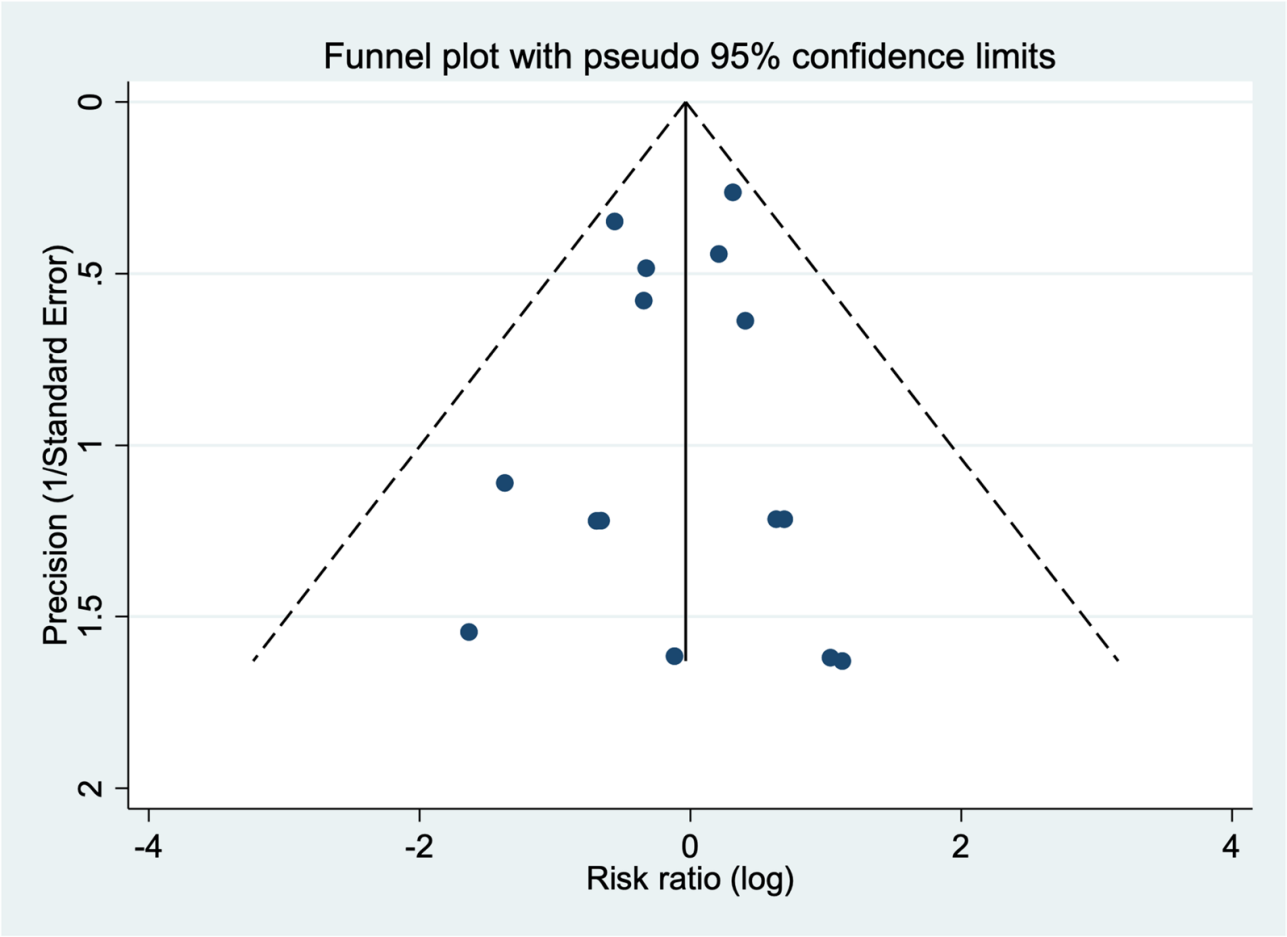
Funnel plot for all-cause mortality

Summary of the subgroup analysis is shown in Table 2. There were no significant differences between the provisional strategy and the two-stent strategy in any of the outcomes across the subgroups of follow-up time and study size. Visual inspection of the funnel plot for the exploratory mortality endpoint did not suggest marked asymmetry, and Egger’s regression test was not statistically significant (p = 0.641). However, this assessment was limited by the small number of available studies and should be interpreted cautiously (Fig. 10).

## Discussion

In this updated meta-analysis of randomized controlled trials, pooled estimates did not demonstrate a statistically significant difference between provisional and two-stent strategies for the exploratory mortality endpoint, cardiac death, myocardial infarction, target lesion revascularization, stent thrombosis, or MACE. These findings should not be interpreted as proof of equivalence or non-inferiority, because the included trials differed in lesion complexity, bifurcation anatomy, stenting technique, follow-up duration, procedural optimization, and endpoint definitions. Publication bias assessment did not suggest small-study effects for the mortality endpoint, but this analysis was limited by the relatively small number of studies available for several outcomes.

The mortality analysis requires cautious interpretation. Because only a minority of trials directly reported all-cause mortality, we used cardiac death when all-cause mortality was unavailable to create an exploratory mortality endpoint. This approach increased the number of analyzable trials but introduced conceptual limitations, as cardiac death is not interchangeable with all-cause mortality and may underestimate non-cardiovascular deaths. Differences in event adjudication and reporting across studies may therefore have influenced the pooled mortality estimate.

Moderate heterogeneity was observed for MI, TLR, and especially MACE. This variability is clinically plausible because the included trials differed in lesion complexity, left main involvement, Medina anatomy, side-branch size and disease length, use of DK-crush, culotte, crush, TAP, or T-stenting techniques, intravascular imaging use, DES generation, final kissing balloon inflation, and proximal optimization practices. In addition, MACE definitions varied across trials, further contributing to methodological heterogeneity. Therefore, pooled results should be interpreted as an overall randomized-trial summary rather than as a definitive comparison of any single two-stent technique against provisional PCI.

Importantly, the present analysis should not be interpreted as showing that all two-stent techniques are equivalent. DK-crush, culotte, crush, TAP, and T-stenting differ procedurally and may have different clinical performance depending on anatomy and operator expertise. Some contemporary trials enriched for complex bifurcation or distal left main disease suggest that selected planned two-stent approaches may be beneficial in anatomically complex lesions. However, the available aggregate data did not permit a sufficiently powered comparison of individual two-stent techniques. Therefore, conclusions regarding the relative performance of specific two-stent methods remain limited.

These findings are broadly consistent with contemporary consensus and guideline recommendations that support provisional stenting as the default strategy for most bifurcation lesions, with planned two-stent PCI reserved for selected complex anatomies, large diseased side branches, or situations in which an acceptable side-branch result is unlikely with a provisional approach [19–21]. A recent systematic review and meta-analysis of ostial LAD bifurcation PCI also highlighted that procedural strategy selection remains anatomy-dependent, reinforcing the need to interpret pooled bifurcation PCI outcomes in the context of lesion location, side-branch involvement, and technique-specific procedural optimization [22]. The present meta-analysis supports an individualized framework but does not directly test procedural optimization steps such as proximal optimization, final kissing balloon inflation, imaging guidance, or physiology-guided side-branch assessment.

In addition to including all relevant randomized controlled trials, this study is most distinguished from its predecessors by the inclusion of the DKCRUSH-V trial, which found that a planned double-kissing crush two-stent technique, compared with a provisional one-stent technique, had decreased target lesion failure at one year but no significant difference in cardiac death between the groups. The weight of this large trial (n = 482) may account for the negative result of this meta-analysis.

Our findings stand at odds with previous meta-analyses, such as Nairooz et al. whose analysis only used data from studies published after 2010 with long-term data and found that a one-stent strategy was associated with reduced all-cause mortality (RR, 0.66, 95% CI 0.45–0.98). These findings are in accord with Behan et al.’s pooled analysis of BBC1 and Nordic 1 studies [12], which similarly found an association between one-stent technique and reduced mortality. Behan et al. noted a separation of all-cause mortality Kaplan–Meier curves at approximately 2 years; therefore, it may be that isolating any advantageous effects of one- over two-stent strategies are apparent only in data that reflect modern stent technology and long-term follow up.

A recent observational sub-study of the EXCEL trial that has been presented but not yet published by Kandzari et al., found that patients who underwent provisional stenting compared to an up-front two-stent strategy had no difference in outcomes at 30 days but reduced composite end point of mortality, myocardial infarction, stroke, or target lesion revascularization at 3 years [23]. While this may represent a long term benefit to provisional stenting, the lack of randomization makes it more likely that the worse outcomes associated with the patients who underwent planned two-stent interventions was caused by the more severe and complex coronary artery disease which led the concerned operators to choose a two-stent strategy.

While the optimal interventional strategy for significant coronary bifurcation disease remains debated, the present synthesis of randomized trial data does not support a generalized preference for one- or two-stent technique across all bifurcation lesions. Further investigation using modern stent platforms, standardized procedural optimization, and long-term follow-up is needed and may clarify whether specific anatomical subgroups benefit from specific two-stent techniques.

### Limitations

This study has several limitations. First, all-cause mortality was not directly reported in all trials, requiring an exploratory mortality endpoint that substituted cardiac death when all-cause mortality was unavailable. The exploratory mortality endpoint may introduce misclassification bias because cardiac death and all-cause mortality are conceptually distinct outcomes. Therefore, the pooled mortality estimate should be interpreted as hypothesis-generating rather than definitive. Second, myocardial infarction and MACE definitions varied across trials, limiting direct comparability. Third, moderate to substantial heterogeneity was observed for myocardial infarction, target lesion revascularization, and MACE, likely reflecting differences in lesion complexity, left main involvement, bifurcation anatomy, stent platforms, procedural optimization, and follow-up duration. Fourth, the two-stent group included multiple techniques, including DK-crush, Culotte, crush, TAP, T-stenting, and mixed approaches, which should not be assumed to have equivalent outcomes. A formal technique-specific subgroup analysis by individual two-stent technique was considered but not performed because the number of trials per technique was small, several trials used mixed or protocol-dependent two-stent approaches, and procedural optimization practices differed substantially across studies. Fifth, publication bias testing was limited by the small number of studies available for several endpoints. Finally, this was an aggregate-data meta-analysis, and individual patient-level analyses would be needed to identify which anatomical or procedural subgroups benefit most from each strategy (Additional file 1).

## Conclusion

The available randomized evidence does not demonstrate clear superiority of either provisional or planned two-stent strategies for major clinical outcomes. However, because included trials differed in lesion complexity, left main involvement, two-stent technique, stent generation, and optimization practices, these findings should not be interpreted as proof of equivalence among all bifurcation PCI strategies. Strategy selection should remain individualized according to anatomy, side-branch disease, procedural complexity, and operator expertise, in line with contemporary consensus recommendations. Future studies should use standardized endpoint definitions, longer follow-up, and adequately powered comparisons of specific two-stent techniques, particularly in complex bifurcation and left main disease.

## Supplementary Information

**Additional file 1: Table S1**. Trial-reported definitions of myocardial infarction and major adverse cardiac events.

## Data Availability

All data relevant to the study are included in the article or uploaded as supplementary information. Corresponding author can also be contacted on email.

## Abbreviations

DES: Drug-eluting stent
DK-crush: Double-kissing crush
EBC: European Bifurcation Club
LM: Left main
POT: Proximal optimization technique
TAP: T-and-protrusion
PCI: Percutaneous coronary intervention
RCT: Randomized controlled trial
CI: Confidence interval
PRISMA: Preferred Reporting Items for Systematic reviews and Meta-Analyses
MACE: Major adverse cardiac events
RR: Relative risk
RoB: Risk of bias
MI: Myocardial infarction
TLR: Target lesion revascularization
ST: Stent thrombosis

## References

[1] Latib A, Colombo A (2008) Bifurcation disease: what do we know, what should we do? JACC Cardiovasc Interv 1(3):218–226 19463303 10.1016/j.jcin.2007.12.008

[2] Louvard Y, Lefèvre T, Morice M-C (2004) Percutaneous coronary intervention for bifurcation coronary disease. Heart 90(6):713–722 15145893 10.1136/hrt.2002.007682 PMC1768265

[3] Garot P, Lefèvre T, Savage M, Louvard Y, Bamlet WR, Willerson JT et al (2005) Nine-month outcome of patients treated by percutaneous coronary interventions for bifurcation lesions in the recent era: a report from the Prevention of Restenosis with Tranilast and its Outcomes (PRESTO) trial. J Am Coll Cardiol 46(4):606–612 16098423 10.1016/j.jacc.2005.01.065

[4] Pan M, de Lezo JS, Medina A, Romero M, Segura J, Pavlovic D et al (2004) Rapamycin-eluting stents for the treatment of bifurcated coronary lesions: a randomized comparison of a simple versus complex strategy. Am Heart J 148(5):857–864 15523318 10.1016/j.ahj.2004.05.029

[5] Colombo A, Moses JW, Morice MC, Ludwig J, Holmes DR, Spanos V et al (2004) Randomized study to evaluate sirolimus-eluting stents implanted at coronary bifurcation lesions. Circulation 109(10):1244–1249 14981005 10.1161/01.CIR.0000118474.71662.E3

[6] Colombo A, Bramucci E, Saccà S, Violini R, Lettieri C, Zanini R et al (2009) Randomized study of the crush technique versus provisional side-branch stenting in true coronary bifurcations: the CACTUS (coronary bifurcations: application of the crushing technique using sirolimus-eluting stents) study. Circulation 119(1):71–78 19103990 10.1161/CIRCULATIONAHA.108.808402

[7] Ferenc M, Gick M, Kienzle R-P, Bestehorn H-P, Werner K-D, Comberg T et al (2008) Randomized trial on routine vs. provisional T-stenting in the treatment of de novo coronary bifurcation lesions. Eur Heart J 29(23):2859–2867 18845665 10.1093/eurheartj/ehn455 PMC2638653

[8] Hildick-Smith D, de Belder AJ, Cooter N, Curzen NP, Clayton TC, Oldroyd KG et al (2010) Randomized trial of simple versus complex drug-eluting stenting for bifurcation lesions: the British Bifurcation Coronary Study: old, new, and evolving strategies. Circulation 121(10):1235–1243 20194880 10.1161/CIRCULATIONAHA.109.888297

[9] Maeng M, Holm NR, Erglis A, Kumsars I, Niemelä M, Kervinen K et al (2013) Long-term results after simple versus complex stenting of coronary artery bifurcation lesions: Nordic bifurcation study 5-year follow-up results. J Am Coll Cardiol 62(1):30–34 23644088 10.1016/j.jacc.2013.04.015

[10] Kim Y-H, Lee J-H, Roh J-H, Ahn J-M, Yoon S-H, Park D-W et al (2015) Randomized comparisons between different stenting approaches for bifurcation coronary lesions with or without side branch stenosis. JACC Cardiovasc Interv 8(4):550–560 25907082 10.1016/j.jcin.2015.01.016

[11] Song YB, Park TK, Hahn J-Y, Yang JH, Choi J-H, Choi S-H et al (2016) Optimal strategy for provisional side branch intervention in coronary bifurcation lesions: 3-year outcomes of the SMART-STRATEGY randomized trial. JACC Cardiovasc Interv 9(6):517–526 27013152 10.1016/j.jcin.2015.11.037

[12] Behan MW, Holm NR, de Belder AJ, Cockburn J, Erglis A, Curzen NP et al (2016) Coronary bifurcation lesions treated with simple or complex stenting: 5-year survival from patient-level pooled analysis of the Nordic Bifurcation Study and the British Bifurcation Coronary Study. Eur Heart J 37(24):1923–1928 27161619 10.1093/eurheartj/ehw170

[13] Hildick-Smith D, Behan MW, Lassen JF, Chieffo A, Lefèvre T, Stankovic G et al (2016) The EBC TWO study (European bifurcation coronary TWO): a randomized comparison of provisional T-stenting versus a systematic 2 stent Culotte strategy in large caliber true bifurcations. Circ Cardiovasc Interv 9(9):e003643 27578839 10.1161/CIRCINTERVENTIONS.115.003643

[14] Chen S-L, Santoso T, Zhang J-J, Ye F, Xu Y-W, Fu Q et al (2017) Clinical outcome of double kissing crush versus provisional stenting of coronary artery bifurcation lesions: the 5-year follow-up results from a randomized and multicenter DKCRUSH-II study (randomized study on double kissing crush technique versus provisional stenting technique for coronary artery bifurcation lesions). Circ Cardiovasc Interv 10(2):e004497 28122805 10.1161/CIRCINTERVENTIONS.116.004497 PMC5319391

[15] Chen S-L, Zhang J-J, Han Y, Kan J, Chen L, Qiu C et al (2017) Double kissing crush versus provisional stenting for left main distal bifurcation lesions: DKCRUSH-V randomized trial. J Am Coll Cardiol 70(21):2605–2617 29096915 10.1016/j.jacc.2017.09.1066

[16] Zimarino M, Corazzini A, Ricci F, Di Nicola M, De Caterina R (2013) Late thrombosis after double versus single drug-eluting stent in the treatment of coronary bifurcations: a meta-analysis of randomized and observational studies. JACC Cardiovasc Interv 6(7):687–695 23769650 10.1016/j.jcin.2013.03.012

[17] Nairooz R, Saad M, Elgendy IY, Mahmoud AN, Habash F, Sardar P et al (2017) Long-term outcomes of provisional stenting compared with a two-stent strategy for bifurcation lesions: a meta-analysis of randomised trials. Heart 103(18):1427–1434 28314731 10.1136/heartjnl-2016-310929

[18] Ford TJ, McCartney P, Corcoran D, Collison D, Hennigan B, McEntegart M et al (2018) Single- versus 2-stent strategies for coronary bifurcation lesions: a systematic review and meta-analysis of randomized trials with long-term follow-up. J Am Heart Assoc 7(11):e008730 29802145 10.1161/JAHA.118.008730 PMC6015365

[19] Neumann FJ, Sousa-Uva M, Ahlsson A, Alfonso F, Banning AP, Benedetto U et al (2019) 2018 ESC/EACTS Guidelines on myocardial revascularization. Eur Heart J 40(2):87–165 30165437 10.1093/eurheartj/ehy394

[20] Lawton JS, Tamis-Holland JE, Bangalore S, Bates ER, Beckie TM, Bischoff JM et al (2022) 2021 ACC/AHA/SCAI guideline for coronary artery revascularization. Circulation 145(3):e18–e114 34882435 10.1161/CIR.0000000000001038

[21] Burzotta F, Lassen JF, Lefèvre T, Banning AP, Chatzizisis YS, Johnson TW et al (2024) Percutaneous coronary intervention for bifurcation coronary lesions using optimised angiographic guidance: the 18th consensus document from the European Bifurcation Club. EuroIntervention 20(7):e478–e493 10.4244/EIJ-D-24-00160 PMC11285041 38752714

[22] Khairy AM, Shafik A, Khaled M, Hussein MA, Elzayat A, Elashry A et al (2025) Comparing cross-over stenting and focal ostial stenting for ostial left anterior descending coronary artery lesions: a systematic review and meta-analysis. BMC Cardiovasc Disord 25:143. 10.1186/s12872-024-04393-x 40000947 10.1186/s12872-024-04393-x PMC11852548

[23] Kandzari D, Gershlick A, Serruys P, Leon M, Morice M-C, Lembo N et al (2019) TCT-83 provisional vs. planned two-stent technique in patients with distal bifurcation left main disease undergoing PCI: the EXCEL trial. J Am Coll Cardiol 70(18 Suppl):B36–B37

