# Supplementary material for: Outcomes in provisional one-stent versus dedicated two-stent coronary bifurcation stenting techniques: a systematic review and meta-analysis
Source: Egypt Heart J. 2026 Jun 16;78:45. doi: 10.1186/s43044-026-00757-0 (PMC13272726; doi:10.1186/s43044-026-00757-0)
Supplement: Supplementary file 1 — Additional file 1: Table S1. Trial-reported definitions of myocardial infarction and major adverse cardiac events. [file 43044_2026_757_MOESM1_ESM.docx]

Supplementary Table 1. Trial-reported definitions of myocardial infarction and major adverse cardiac events

Definitions are summarized from the published trial reports as available. When exact diagnostic criteria were not fully specified in the publication, outcomes were extracted according to the trial-defined endpoint reported by the original investigators. NR indicates not reported or not used as a pooled endpoint in the present analysis.

| **Study** | **MI definition/extraction approach** | **MACE/composite endpoint definition/extraction approach** | **Notes for pooling** |
| --- | --- | --- | --- |
| Pan et al. 2004 (Am Heart J) | Trial-reported myocardial infarction. | Trial-reported composite major adverse cardiac events, including cardiac death, myocardial infarction, stent thrombosis, or target vessel/lesion revascularization as reported. | Used trial-defined endpoints. |
| Colombo et al. 2004 (Circulation) | Trial-reported myocardial infarction. | Trial-reported major adverse cardiac events/composite clinical endpoint as reported in the original publication. | Used trial-defined endpoints. |
| CACTUS / Colombo et al. 2009 | Trial-reported myocardial infarction. | Trial-reported MACE as reported in the original publication. | Included in pooled MACE endpoint when available. |
| Ferenc et al. 2008 T-stenting trial | Trial-reported myocardial infarction. | Trial-reported MACE as reported in the original publication. | Included in pooled MACE endpoint when available. |
| BBC ONE / Hildick-Smith et al. 2010 | Trial-reported myocardial infarction. | Trial-reported MACE/composite outcome as reported in the original publication. | Used trial-defined endpoints; definitions may differ from other trials. |
| Nordic Bifurcation Study / Maeng et al. 2013 | Trial-reported myocardial infarction. | Composite endpoint reported as cardiac death, myocardial infarction, stent thrombosis, or target vessel revascularization/target lesion revascularization depending on the report. | Used trial-defined endpoint. Hypercholesterolemia values in Table 1 were checked as 78% vs 72%. |
| EBC TWO / Hildick-Smith et al. 2016 | Trial-reported myocardial infarction. | Trial-reported MACE/composite endpoint as reported in the original publication. | Used trial-defined endpoints. |
| SMART-STRATEGY / Song et al. 2016 | Trial-reported myocardial infarction. | Composite endpoint reported by the trial; extracted as trial-defined endpoint. | Used trial-defined endpoints. |
| CROSS / Kim et al. 2015 | Trial-reported myocardial infarction. | Trial-reported MACE/composite endpoint as reported in the original publication. | Used trial-defined endpoints. |
| PERFECT / Kim et al. 2015 | Trial-reported myocardial infarction. | Trial-reported MACE/composite endpoint as reported in the original publication. | Used trial-defined endpoints. |
| DKCRUSH-II / Chen et al. 2017 | Trial-reported myocardial infarction. | Trial-reported composite endpoint as reported by the original investigators. | Used trial-defined endpoints. |
| DKCRUSH-V / Chen et al. 2017 | Target-vessel myocardial infarction reported as part of the trial endpoint; extracted according to the original report. | Primary trial endpoint was target lesion failure, defined as cardiac death, target-vessel myocardial infarction, or clinically driven target lesion revascularization. | TLF was not treated as identical to MACE unless matched to the outcome used in the pooled analysis. |
| DEFINITION II / Zhang et al. 2020 | Trial-reported myocardial infarction. | Trial-reported target lesion failure/composite endpoint as defined in the original publication. | Used trial-defined endpoints. |
| EBC MAIN / Hildick-Smith et al. 2021 | Trial-reported myocardial infarction. | Trial-reported composite clinical endpoint as defined in the original publication. | Used trial-defined endpoints; kissing balloon data in Table 1 reflect the original report. |
| Crossover vs Side-Branch Opening / Lee et al. 2021 | Trial-reported myocardial infarction. | NR for pooled MACE endpoint unless reported in the original publication in a compatible format. | Used trial-defined endpoints where available. |
